# Anaesthetic induction and recovery characteristics of a diazepam-ketamine combination compared with propofol in dogs

**DOI:** 10.4102/jsava.v86i1.1258

**Published:** 2015-06-01

**Authors:** Jacques P. Ferreira, T. Brighton Dzikiti, Gareth E. Zeiler, Roxanne Buck, Bruce Nevill, Bruce Gummow, Lynette Bester

**Affiliations:** 1 Department of Companion Animal Clinical Studies, University of Pretoria, South Africa; 2 College of Public Health, Medical and Veterinary Sciences, James Cook University, Australia

## Abstract

Induction of anaesthesia occasionally has been associated with undesirable behaviour in dogs. High quality of induction of anaesthesia with propofol has been well described while in contrast variable induction and recovery quality has been associated with diazepam-ketamine. In this study, anaesthetic induction and recovery characteristics of diazepam-ketamine combination with propofol alone were compared in dogs undergoing elective orchidectomy. Thirty-six healthy adult male dogs were used. After habitus scoring (simple descriptive scale [SDS]), the dogs were sedated with morphine and acepromazine. Forty minutes later a premedication score (SDS) was allocated and general anaesthesia was induced using a combination of diazepam-ketamine (Group D/K) or propofol (Group P) and maintained with isoflurane. Scores for the quality of induction, intubation and degree of myoclonus were allocated (SDS). Orchidectomy was performed after which recovery from anaesthesia was scored (SDS) and times to extubation and standing were recorded. Data were analysed using descriptive statistics and Kappa Reliability and Kendall Tau B tests. Both groups were associated with acceptable quality of induction and recovery from anaesthesia. Group P, however, was associated with a poorer quality of induction (*p* = 0.014), prolonged induction period (*p* = 0.0018) and more pronounced myoclonus (*p* = 0.003), but had better quality of recovery (*p* = 0.000002) and shorter recovery times (*p* = 0.035) compared with Group D/K. Diazepam-ketamine and propofol are associated with acceptable induction and recovery from anaesthesia. Propofol had inferior anaesthetic induction characteristics, but superior and quicker recovery from anaesthesia compared with diazepam-ketamine.

## Introduction

The combination of diazepam and ketamine is a commonly described protocol for induction of general anaesthesia in healthy dogs of various ages. It may also be indicated in certain cases with cardiovascular compromise (Beteg *et al.* 2010; Boutureira, Trim & Cornell 2007; Fayyaz *et al.* 2009; Green *et al.* 1981; Haskins, Farver & Patz 1986; Hazra, De & Roy 2008; Hellyer, Freeman & Hubbell 1991; Kolata 1986; White, Shelton & Taylor 2001). The combination of diazepam and ketamine, at a dose range of 0.2 mg/kg – 0.5 mg/kg and 5 mg/kg – 10 mg/kg respectively, has generally been associated with excitement-free induction of anaesthesia in dogs. However, maintenance of pharyngeal and laryngeal reflexes as well as hypersalivation have resulted in difficult intubations being reported (Green *et al.* 1981; Hellyer *et al.* 1991; White *et al.* 2001). Recovery from diazepam-ketamine in dogs has been reported to be free of emergence excitation although it is commonly associated with ataxia (Beteg *et al.* 2010; White *et al.* 2001).

Propofol (2, 6-di-isopropylphenol) has a wide initial dose range (2 mg/kg – 8 mg/kg) in dogs and induces rapid central nervous system depression facilitating anaesthetic induction within 20–30 seconds after commencement of intravenous administration (Robinson & Borer-Weir 2013; Watkins, Hall & Clarke 1987). Propofol bypasses the early stages of anaesthetic depth that are often associated with involuntary clonic movements, thereby facilitating routine excitement-free anaesthetic inductions in up to 92.5% of cases (Branson 2007; Davies 1991; Glowaski & Wetmore 1999). However, adverse induction behaviour characterised by paddling of limbs, muscle twitches and pain on injection has previously been associated with propofol administration in dogs (Davies 1991; Smith *et al.* 1993).

Recovery from propofol is rapid and excitement free, with return to consciousness occurring approximately 20 minutes after bolus administration (Branson 2007). Poor recoveries from propofol are uncommon and are characterised by tremors, opisthotonus, excessive salivation and vomiting (Robertson, Johnston & Beemsterboer 1992; Smith *et al.* 1993). Diazepam-ketamine induction and recovery characteristics compared with propofol have not been reported previously. The aim of this prospective clinical study was to compare a diazepam-ketamine combination with propofol alone for anaesthetic induction and recovery.

## Research method and design

Thirty-six healthy male small-breed dogs weighing a mean (± s.d.) of 5.5 kg ± 2.3 kg and with a mean age of 26 ± 13 months were randomly assigned to an induction regimen of either diazepam-ketamine (Group D/K) or propofol (Group P). Dogs were declared healthy based on physiologically normal haematological (haematocrit, total serum protein, blood smear) and serum chemistry (blood urea nitrogen, serum creatinine) profiles and clinical examination performed upon admission to the hospital.

### Experimental design and procedure

Prior to anaesthesia, each dog was starved of food for 8–12 hours, then placed in a quiet, warm cage and left undisturbed for 30 minutes prior to being allocated a cage habitus score (simple descriptive score, SDS; Table 1) by the primary investigator. A pre-anaesthetic medication of acepromazine (ACP) (Neurotranq®, Virbac, South Africa) at 0.02 mg/kg and morphine (Morphine sulphate Fresenius PF, Fresenius Kabi, South Africa) at 0.3 mg/kg was then administered intramuscularly to all dogs, followed by allocation of a sedation score 40 minutes later (SDS; Table 1).

**TABLE 1:** Simple descriptive scale for scoring habitus during cage rest, sedation following premedication (acepromazine 0.02 mg/kg and morphine 0.3 mg/kg) intramuscularly and quality of intravenous induction, intubation and incidence of myoclonus,with either propofol or diazepam-ketamine in male dogs.

Criteria scored	Score	Description
Cage habitus	0	Severely anxious and aggressive, vocalising, no body tremors
	1	Anxious and vocalising, no body tremors
	2	Anxious but responsive to external stimuli
	3	Calm and responsive to external stimuli
Sedation†	0	No change from pre-sedation behaviour
	1	Slight sedation, still able to walk
	2	Ataxic and heavily sedated, able to walk
	3	Very heavily seated, unable to walk
Quality of induction‡	0	Calm transition, no paddling
	1	Occasional, slow paddling movements
	2	Moderate, sustained paddling movements
	3	Marked paddling, struggling or vocalisation
Intubation	0	Easy intubation
	1	Mild coughing
	2	Pronounced coughing
	3	Swallowing, coughing and gagging
Myoclonus‡	0	No twitching
	1	Occasional, mild muscle twitching
	2	Moderate, sustained muscle twitching
	3	Severe muscle twitching with opisthotonus and/or extensor muscle rigidity

*Source*: Adapted from Amengual, M., Flaherty, D., Auckburally, A., Bell, A.M., Scott, E.M. & Pawson, P., 2013, ‘An evaluation of anaesthetic induction in healthy dogs using rapid intravenous injection of propofol or alfaxalone’, *Journal of Veterinary Anaesthesia and Analgesia* 40(5), 115–123. http://dx.doi.org/10.1111/j.1467-2995.2012.00747.x

†, Dogs exposed to call and clapping, dog then walked from cage to preparation room approximately 2 metres away; ‡, Assessed from commencement of administration of induction protocol until initiation of isoflurane administration.

Immediately after sedation scoring, a 22-gauge indwelling cannula (Jelco®, Smiths Medical, South Africa) was inserted into the right cephalic vein to facilitate intravenous administration of anaesthetic induction drugs. General anaes­thesia was induced in Group-D/K dogs with an initial combination dose of diazepam (A-Lennon Diazepam 0.5%, Aspen Pharmacare, South Africa) and ketamine (Ketamine Fresenius 10%, Fresenius Kabi, South Africa) of 0.375 mg/kg and 5 mg/kg, respectively and in Group-P dogs with an initial propofol (Propofol Fresenius 1%, Fresenius Kabi, South Africa) dose of 2 mg/kg. Induction boli were administered over a 30-second period using a volumetric infusion pump (Perfusor^®^, B Braun, Germany). Sixty seconds after initiation of bolus administration, depth of anaesthesia was assessed by a co-investigator by orderly testing of the lateral and medial palpebral reflex, menace reflex and jaw tone. If depth of anaesthesia was deemed insufficient for endotracheal intubation, a follow-up intravenous bolus was administered, which in Group-D/K dogs was 0.175 mg/kg and 2.5 mg/kg of diazepam and ketamine, respectively and in Group-P dogs was 1 mg/kg of propofol over 30 seconds. Follow-up boli were administered until endotracheal intubation could be achieved.

Endotracheal intubation was performed by the same co-investigator throughout the study using a cuffed, correctly sized, polyvinyl chloride endotracheal tube that was subsequently connected to a Mapleson F breathing system (infant T-piece breathing system, Intersurgical, United Kingdom). The system delivered isoflurane (Isofor®, Safeline Pharmaceuticals, South Africa) in oxygen via a Tec5 out-of-circuit precision vaporiser (Vetequip, USA) initially set to 2% with a fresh gas flow rate set to 600 mL/kg/min. Spontaneous ventilation was permitted.

The overall quality of anaesthetic induction was then scored according to three separate induction criteria as described in Table 1 by the primary investigator. In addition to the scores allocated, total induction doses were calculated per dog and time to induction of anaesthesia was recorded. The target surgical area was aseptically prepared for the orchidectomy, which was performed in a designated theatre by the same specialist surgeon. All anaesthetised dogs were monitored continuously, with heart rate, respiratory rate, oscillometric blood pressure and peripheral oxygen saturation of haemoglobin (S_p_O_2_) measured and recorded every 5 minutes. A balanced crystalloid (Ringer's Lactate, Fresenius Kabi, South Africa) was administered intra-operatively at a rate of 10 mL/kg/h for the duration of the anaesthesia.

Surgery time was recorded on completion of the orchidectomy, isoflurane and oxygen administration stopped and the dogs moved to a designated recovery area to recover from general anaesthesia. A quality of recovery score was then allocated by the primary and co-investigator (Table 2).

**TABLE 2:** Scoring system used for quality of recovery† from anaesthesia in dogs induced with either diazepam-ketamine or propofol and maintained on ­isoflurane.

Category	Description
1	Early – Extubated, calm transition to alertness, coordinated movement, calm
	Late – Alert, coordinated movement, calm
2	Early – Fairly calm transition, holds head up, no body movement attempted
	Late – Holds head up, no body movement
3	Early – Unremarkable transition, routine extubation, some incoordination, does not startle, generally quiet
	Late – Some uncoordinated movements, generally very quiet
4	Early – Unremarkable transition, routine extubation, limited muscle control, startles, may paddle or whine
	Late – Uncoordinated whole body movement, startles, vocalises
5	Early – Struggling during transition, difficult extubation with chewing and coughing elicited, uncoordinated whole body movements, startles, vocalises
	Late – Uncoordinated whole body movements, startles, vocalises
6	Early – Violent transition, restraint required for extubation, emergence delirium, thrashing, cannot be restrained easily
	Late – Emergence delirium, thrashing, cannot be restrained easily

*Source*: Adapted from Jiménez, C.P., Mathis, A., Mora, S.S., Brodbelt, D. & Alibhai, H., 2012, ‘Evaluation of the quality of the recovery after administration of propofol or alfaxalone for induction of anaesthesia in dogs anaesthetized for magnetic resonance imaging’, *Journal of* *Veterinary Anaesthesia and Analgesia* 39(2), 151–159. http://dx.doi.org/10.1111/j.1467-2995.2011.00678.x

†, Observed from termination of isoflurane anaesthesia onwards.

Video recording of the entire induction protocol was performed for retrospective analysis by a specialist anaesthetist blinded to the induction agents used. The same induction scoring system (Table 1) was used for the retrospective assessment.

### Statistical analysis

The collected data were divided into Group D/K and Group P for comparison and tested for normality using histogram analyses. Parametric data (age, weight, anaesthetic maintenance period, extubation time and time to standing) were analysed for statistical significance using the *t*-test, while non-parametric data (all scores) were analysed using the Wilcoxon-Mann-Whitney test. A significant level of *p* < 0.05 was set. Comparison of agreement between the observers was tested using the Kappa Reliability or Kendall Tau B tests (Landis & Koch 1977).

## Results

No statistically significant differences (*p* > 0.05) between Group D/K and Group P were observed with regard to age and weight. Additionally, there were no statistically significant differences with regard to cage habitus score, sedation score and duration of anaesthetic maintenance (Table 3).

**TABLE 3:** Peri-trial observations on dogs presented for orchidectomy in which anaesthesia was induced with either diazepam-ketamine (Group D/K, *n* = 18) or propofol (Group P, *n* = 18) prior to maintenance with isoflurane.

Observation	Group D/K	Group P
Age (months)	24 ± 14	26 ± 12
Weight (kg)	6.4 ± 2.1	4.9 ± 2.2
Cage rest score	2 (0–3)	2 (0–3)
Sedation score	1 (0–3)	1 (0–1)
Total induction dose (mg/kg)†	D: 0.56 ± 1.18	P: 5.94(1.9–0)
	K:7.36 ± 0.14	P: 5.94(1.9–0)
Number of follow-up boli‡	1 (0–2)	3 (0–8)
Intubation score	2 (0–3)	2 (0–3)
Myoclonus score	0 (0–1)	1 (0–3)
Anaesthetic Induction period (minutes)§	3.1 ± 1.1*	6.5 ± 3.9*
Anaesthetic maintenance period (minutes)¶	31.0 ± 4.7	32.3 ± 8.8
Extubation time (minutes)††	9.6 ± 3.7	9.7 ± 3.4
Time to standing (minutes)††	19.0 ± 4.2*	13.7 ± 3.7*

Note: Data expressed as mean and standard deviation or median and range where applicable.

†, Average induction dose to achieve intubation; ‡, Average number of follow-up boli required to achieve intubation; §, Time recorded from commencement of induction agent(s) administration until intubation achieved; ¶, Measured from commencement of isoflurane administration until termination of administration; ††, Time recorded from termination of isoflurane administration.

D, diazepam; K, ketamine; P, propofol.

*, *p* < 0.05.

**FIGURE 1:**
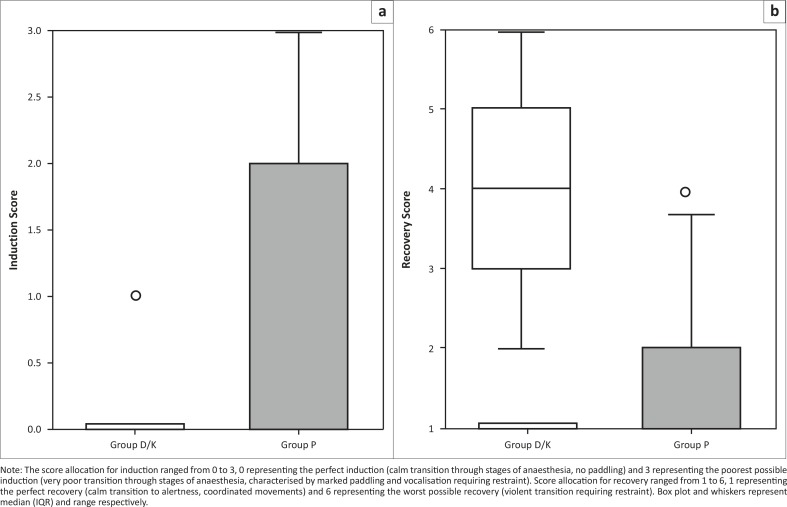
Comparison of (a) induction and (b) recovery scores (simple descriptive scale) of intact male dogs anaesthetised with propofol (Group P) or diazepam-ketamine (Group D/K) for orchidectomy.

The difference in induction score between the two groups, however, was statistically significant (*p* = 0.014), as Group D/K had better induction scores depicted by shorter induction times (*p* = 0.018) and fewer follow-up boli required to achieve endotracheal intubation (Table 3, Figure 1). Clinically, however, both groups produced acceptable anaesthesia, with only seven dogs in Group P and one dog in Group D/K failing to score perfectly (exhibited varying amounts of paddling). The latter was an outlier (inferior score) (Figure 1). No statistically significant differences were observed with regard to intubation score.

Group P had a greater incidence of myoclonus than Group D/K (*p* = 0.003), with nine dogs in Group P (*n* = 18) observed to have myoclonus. Group D/K had a very low incidence of myoclonus, with only one dog having muscle tremors.

There was a statistically significant difference in scores for quality of recovery from anaesthesia between the groups (*p* = 0.00002) and Group P had significantly superior recoveries when compared with Group D/K (Figure 1). Group-D/K dogs had inferior recoveries from anaesthesia, with 15 of the 18 dogs showing ataxia of varying degrees (7 of the 15 evinced paddling on recovery). One dog in Group P was an outlier on recovery. There was no statistically significant difference in time from termination of isoflurane to extubation of the patients in Group P and Group D/K, but time to standing was significantly shorter in Group P (*p =* 0.035; Table 3). Clinically, however, both Group P and Group D/K had generally acceptable recovery from anaesthesia. No dogs in Group P and only seven dogs in Group D/K scored higher than 4 and all dogs were able to stand 23 minutes after termination of isoflurane administration.

The level of agreement between observers was moderate to substantial (0.40–0.75) in all categories of induction and recovery scoring, barring the intubation score, where Kendall's Tau B test indicated fair agreement (0.32).

## Ethical considerations

All dogs were client owned and consent was required in writing prior to being enrolled in the study. The dogs enrolled in the study were exposed to a moderate degree of discomfort as a result of the orchidectomy performed. The surgical procedure was performed by a specialist surgeon and all venepunctures as well as intravenous cannula placement for blood collection and drug administration, respectively, were performed by experienced veterinarians to limit the level of discomfort experienced. Appropriate analgesia including morphine and carprofen were provided during the peri-anaesthetic period. The present study was pre-approved by both the Animal Ethics Committee and the Research Committee of the Faculty of Veterinary Science, University of Pretoria (V017-33).

## Discussion

This study demonstrated that Group D/K was associated with better quality of induction and myoclonus scores when compared with Group P. Recovery from anaesthesia was observed to be inferior and of longer duration for Group D/K than for Group P.

The high quality of induction score associated with Group D/K in the present study supports current literature, which describes excitement-free dissociative anaesthesia with sufficient muscle relaxation to permit endotracheal intubation in dogs (Beteg *et al.* 2010; Hellyer *et al.* 1991; White *et al.* 2001). Maintenance of pharyngeal and laryngeal reflexes is expected with dissociative anaesthesia and although difficult endotracheal intubations have been reported previously, adequate doses (diazepam: 0.5 mg/kg – 1.0 mg/kg; ketamine: 5 mg/kg – 10 mg/kg) of both agents as described in the present study facilitated simple endotracheal intubation, favourable intubation scores and short induction times (Haskins *et al.* 1986; Jackson *et al.* 2004; White *et al.* 2001).

The observation of inferior quality of induction scores associated with Group P in the present study when compared with Group D/K was surprising and contrary to published literature (Amengual *et al.* 2013; Bufalari *et al.* 1998; Hall & Chambers 1987). The present study's design involved the comparison of two commonly used clinical protocols under clinical conditions. Statistically the quality of induction with propofol was inferior to diazepam-ketamine; however, both were clinically acceptable, demonstrating desirable anaesthetic induction characteristics in the majority of dogs anaesthetised.

Time to induction of anaesthesia in Group P was statistically longer and required more follow-up boli to achieve endotracheal intubation when compared with Group D/K. These findings are contrary to those of Watkins *et al.* (1987) and Amengual *et al.* (2013), who described rapid induction of anaesthesia facilitating easy endotracheal intubation in dogs anaesthetised with propofol administered at similar doses. Possible explanations for the induction characteristics associated with Group P include the following:

- induction technique (induction dose and rate of administration)
- degree of pre-anaesthetic sedation achieved
- clinician experience
- scoring system used
- signalment of the dogs that were used in the trial.

The propofol dose range described for anaesthetic induction in dogs is wide (Jiménez *et al.* 2012; Robinson & Borer-Weir 2013). The initial induction dose in the present study (2 mg/kg) reflected the minimum induction dose described by Robinson & Borer-Weir and the mean propofol induction dose observed in the study of 6 mg/kg was comparable to doses reported by Doebeli *et al.* (2013). The rate of propofol administration may influence the cardiorespiratory system, potentially resulting in hypotension and apnoea if administered rapidly (Keates & Whittem 2012). This was not the case in the present study. Conversely, in the present study propofol was administered at a rate of 2 mg/minute, half the dose rate reported by Bufalari *et al.* (1998) and Robinson and Borer-Weir *et al.* (2013). The slow rate of propofol administration may have resulted in partial anaesthetic recovery characterised by paddling, requiring numerous follow-up boli and delayed endotracheal intubation (Branson 2007; Musk *et al.* 2005; Zoran, Riedsel & Dyer 1993). As a result, fair comparison of induction, intubation and myoclonus scores of Group P to Group D/K is limited.

Adequate premedication provides anxiolysis, muscle relaxation and analgesia as well as decreasing induction agent dose requirements (Grint, Alderson & Dugdale 2010). Pre-anaesthetic administration of ACP and morphine is commonly utilised in dogs and produces mild to moderate sedation throughout a wide dose range (Brodbelt, Taylor and Stanway 1997; Grint *et al.* 2010; Henao-Guerrero & Riccó 2014; Heard, Webb & Daniels 1986; Monteiro *et al.* 2009; Robertson *et al.* 2001; Smith *et al.* 2001). The dose of ACP (0.02 mg/kg) and morphine (0.3 mg/kg) used in the present study were conservative and resulted in a light degree of sedation as demonstrated by the low sedation scores. Had there been more pronounced sedation, it is likely that the induction dose as well as the rate of administration used in the propofol group would have been adequate to facilitate easy endotracheal intubation as described by Amengual *et al.* (2013). In addition, this would have resulted in shorter and excitement-free anaesthetic inductions characterised by less myoclonus (Musk *et al.* 2005).

Timeous endotracheal intubation after induction of anaesthesia with a short-acting induction agent may be challenging, as previously reported in dogs anaesthetised with propofol (Clarke & Hall 1990; Davies 1991). Robinson and Borer-Weir (2013) reported that prolonged intubation times and a greater number of follow-up boli of induction agent were required when intubations were performed by inexperienced personnel. In this study, assessment of anaesthetic depth and subsequent intubation were performed by a relatively inexperienced clinician, potentially contributing to the inferior induction and intubation scores obtained in Group P.

Signalment plays a role in the induction dose requirements in dogs. A study performed by Boveri, Brearley and Dugdale (2013) highlighted the importance of calculating propofol dose requirements based on lean body mass. Body condition score was not assessed during the pre-anaesthetic clinical examination, which is a limitation in the present study as it may have provided a more accurate dose requirement for the dogs induced with propofol.

One dog in Group D/K that scored poorly on induction and, similar to an outlier in the study by White *et al.* (2001), may have been given insufficient pre-anaesthetic sedation.

Recovery was statistically superior and shorter in Group P when compared with Group D/K. Historically propofol generally has been associated with acceptable recoveries and the results of the present study further support published literature (Bufalari *et al.* 1998; Smith *et al.* 1993; Suarez *et al.* 2012). One dog in Group P, however, did receive an inferior recovery score, evincing muscle tremors, inability to maintain sternal recumbency and vocalisation. Propofol previously has been associated with myoclonus, paddling and opisthotonus during the recovery period, although vocalisation has not been generally reported (Davies 1991; Robertson *et al.* 1992; Tsai, Wang & Yeh 2007).

Dogs induced with diazepam-ketamine were associated with statistically inferior and prolonged recoveries when compared with propofol. Clinically, however, the quality of anaesthesia was acceptable. Recovery from diazepam-ketamine demon­strated an unremarkable return to cons­ciousness, routine extubation, occasional paddling and vocalisation. Such a recovery, in a clinical setting, is considered acceptable as dogs were not at risk of self-inflicted injury as a result of trauma and most dogs would be able to stand after a relatively short period. Subjectively superior recoveries when compared with the present study's results were described by White *et al.* (2001) in dogs recovering from anaesthesia induced with diazepam-ketamine and maintained on isoflurane for 103 ± 43 minutes. Compared with that study, the present study had a relatively short duration of anaesthetic maintenance (31.3 ± 8 minutes). The shorter duration may not have provided sufficient time for metabolism and redistribution to significantly decrease the diazepam-ketamine plasma concentration prior to commencement of anaesthetic recovery, resulting in subjectively poorer recovery quality being observed (Jiménez *et al.* 2012).

The subjective scoring systems incorporated in the present study successfully differentiated the quality of induction and recovery between two groups of healthy dogs. A moderate to good level of agreement between observers was achieved but only after sufficient training on the correct use of the scales. The SDS scoring systems used to score induction and recovery from anaesthesia have been described previously in literature but have not been fully validated (Amengual *et al.* 2013; Jiménez *et al.* 2012). Additionally, the validity of the SDS scores used in the study by Jiménez *et al.* was recently questioned by Ferchichi *et al.* (2013) in terms of:

- the methodology of associating induction and recovery characteristics to a single agent without confirming adequate plasma concentrations of the induction agent/s
- the strength of comparing induction scores from two induction agents where administrations of the agents were not at equipotent doses
- remarking on the quality of anaesthetic recovery from a specific induction agent without demonstrating the presence of the drug in adequate concentrations in plasma.

These arguments raised valid concerns and demonstrate limitations in the present study as well as the study performed by Jiménez *et al.* (2012). The results in the present study, however, are still relevant in the clinical setting, where subjectively assessed anaesthetic depth provides the end-point for titration of induction agents prior to intubation. Similarly, the inferior recovery scores consistently associated with diazepam-ketamine during the recovery period in the present study when compared with the near perfect recoveries observed in dogs induced with propofol remain clinically relevant. Separate score allocations for the period prior to extubation (early phase) and the period from extubation to standing (late phase) may have improved the accuracy of evaluating recovery quality and would be useful additions to follow-up research.

### Limitations of the study

The present study performed anaesthesia exclusively in healthy, adult male dogs weighing less than 10 kg. Future studies performed in female dogs, juvenile dogs or dogs weighing in excess of 10 kg may yield different results and outcomes.

## Conclusion

The use of propofol alone and a diazepam-ketamine combination both produced clinically acceptable induction of anaesthesia in the dogs in this study; however, propofol administered at a low dose rate produced measurably inferior induction characteristics. Recovery from anaesthesia induced with both of these protocols was satisfactory; however, recovery from propofol was more rapid and associated with less excitement and ataxia.
